# Transcatheter Arterial Embolization for Bleeding Caused by Endoscopic Ultrasound-Guided Fine-Needle Aspiration: A Case Series

**DOI:** 10.7759/cureus.55025

**Published:** 2024-02-27

**Authors:** Yasuyuki Onishi, Hironori Shimizu, Shintaro Kimura, Shojiro Oka, Seiya Kawahara, Norimitsu Uza, Hiroyoshi Isoda, Yuji Nakamoto

**Affiliations:** 1 Diagnostic Imaging and Nuclear Medicine, Kyoto University, Kyoto, JPN; 2 Diagnostic Radiology, National Cancer Center Hospital, Tokyo, JPN; 3 Diagnostic Radiology, Kobe City Medical Center General Hospital, Kobe, JPN; 4 Radiology, Otsu Red Cross Hospital, Otsu, JPN; 5 Gastroenterology and Hepatology, Kyoto University, Kyoto, JPN

**Keywords:** endoscopic ultrasound-guided fine-needle aspiration, pancreas, transcatheter arterial embolization, bleeding, complication

## Abstract

Introduction

Bleeding is the most frequent complication of endoscopic ultrasound-guided fine-needle aspiration (EUS-FNA). In a few cases of massive bleeding caused by EUS-FNA, transcatheter arterial embolization (TAE) has been used to obtain hemostasis. We present a case series of patients who underwent TAE for bleeding due to EUS-FNA.

Methods

This case series included six patients (five men and one woman) who underwent TAE for bleeding caused by EUS-FNA between January 2018 and December 2022 at the four institutions involved in this study. The median age at TAE was 72.5 years (range, 67-83 years). The target sites for EUS-FNA were the pancreatic tail (n = 3), pancreatic head (n = 2), and hepatic hilar lymph nodes (n = 1). The angiographic findings, embolization procedures, technical and clinical success rates, and TAE complications were retrospectively assessed.

Results

Angiography revealed contrast-media extravasation or pseudoaneurysms in five patients. In all patients, TAE using a microcatheter was performed via the transfemoral approach. N-butyl cyanoacrylate, coils, and gelatin sponges were used for embolization. The technical and clinical success rates of TAE were 100%. One complication, a duodenal ulcer, developed in one patient and was managed conservatively.

Conclusion

TAE is an effective and safe treatment for EUS-FNA-induced bleeding.

## Introduction

Endoscopic ultrasound-guided fine-needle aspiration (EUS-FNA) is a widely accepted, safe, and useful procedure for the diagnosis of various lesions [1-3]. The complications associated with EUS-FNA include bleeding, gastrointestinal perforation, peritonitis, infection, acute pancreatitis, and needle-tract seeding, with bleeding being the most frequent complication [4]. Bleeding is self-limiting in most cases, and endoscopic hemostasis and blood transfusions are rarely needed [4]. However, in a few cases of massive bleeding caused by EUS-FNA, transcatheter arterial embolization (TAE) has been reportedly used [5,6]. Here, we present a case series of six patients who underwent TAE for bleeding due to EUS-FNA.

## Materials and methods

Patients

The Ethics Committee of Kyoto University Graduate School and Faculty of Medicine granted approval for this case series (approval no. R3995). The requirement for informed consent was waived due to the retrospective nature of the study. We collected data from the four institutions involved in the study: Kyoto University Hospital, Kyoto; National Cancer Center Hospital, Tokyo; Kobe City Medical Center General Hospital, Kobe; and Otsu Red Cross Hospital, Otsu. During the study period (between January 2018 and December 2022), 4217 EUS-FNA procedures were performed at the gastroenterology departments of the four institutions. Six patients out of these cases underwent TAE for bleeding from EUS-FNA. Cases of bleeding caused by EUS-FNA-induced pancreatitis were excluded, as the effectiveness of TAE for bleeding caused by pancreatitis has been reported previously [7,8]. Of the six patients, five were male and one female, with a median age of 72.5 years (range, 67-83 years). Three patients were administered antithrombotic agents; two patients were administered aspirin, which was discontinued on the day of the EUS-FNA; and one patient was administered aspirin and clopidogrel, which were discontinued six and 10 days before the EUS-FNA, respectively. In all patients, EUS-FNA was performed to obtain tissue samples from a mass lesion for diagnosis. In five patients, the EUS-FNA target was in the pancreas. The median needle size for EUS-FNA was 22 gauge (range, 19-22) and the median number of needle passes in EUS-FNA was 2 (range, 2-4). Bleeding was observed during EUS-FNA in four patients. In one patient, bleeding was managed by compression using an EUS probe. In another patient, bleeding was managed by endoscopic clipping. In the remaining two patients with bleeding, the hematoma did not enlarge during the procedure, and no treatment was needed to obtain hemostasis. In the patient who underwent clipping for bleeding, contrast-enhanced CT was used to evaluate bleeding immediately after EUS-FNA. The remaining five patients had abdominal pain and anemia on the same day or within several days of EUS-FNA, and contrast-enhanced CT was performed. The CT revealed a hematoma around the biopsy-targeted mass in all patients. Contrast-media extravasation or pseudoaneurysms were observed in five patients. In all patients, emergency TAE was performed based on CT findings. The median period between EUS-FNA and TAE was one day (range, zero to seven days). The patient characteristics are presented in Table 1.

**Table 1:** Patient characteristics CKD: chronic kidney disease; CT: computed tomography; EUS-FNA: endoscopic ultrasound-guided fine-needle aspiration; TAE: transcatheter arterial embolization ⁎In this patient, CT was performed immediately after EUS-FNA to evaluate bleeding. †In this patient, CT was performed twice before TAE. Although contrast-media extravasation or a pseudoaneurysm was not detected, a second CT scan performed three hours later revealed enlargement of the hematoma, and TAE was performed.

Cases	Age (years)/ Sex	Comorbidities	Target of EUS-FNA	Pathological diagnosis	Bleeding during EUS-FNA	Signs and symptoms before CT	CT findings	Time from EUS-FNA to TAE (days)
1	67/M	CKD, angina pectoris	Pancreatic tail mass	Adenocarcinoma	Bleeding was managed with clipping	None⁎	No extravasation or pseudoaneurysm†	0
2	71/M	None	Hepatic hilar lymph node	Peripheral T-cell lymphoma	No	Abdominal pain	Extravasation	1
3	71/F	None	Pancreatic head mass	Neuroendocrine tumor	Hematoma was observed in the pancreatic head mass. Hematoma was stable during the procedure.	Abdominal pain, anemia	Pseudoaneurysm	4
4	74/M	Diabetes mellitus	Pancreatic tail mass	Adenocarcinoma	Hematoma was observed between the stomach and the pancreas. Hematoma was stable during the procedure	Abdominal pain, hypotension	Extravasation	0
5	81/M	None	Pancreatic tail mass	Adenocarcinoma	Bleeding was managed with compression by the EUS probe	Abdominal pain	Pseudoaneurysm	7
6	83/M	CKD, cerebrovascular disease	Pancreatic head mass	No tumor	No	Anemia	Extravasation	1

Evaluation

Angiography findings and embolization procedures were recorded in all patients. In cases with contrast-media extravasation or pseudoaneurysm observed on angiography, the technical success of TAE was defined as the absence of these findings on postembolization angiography. In the absence of contrast-media extravasation or pseudoaneurysms, the target artery for embolization was selected based on computed tomography (CT) findings, and technical success was defined as stasis of blood flow in the target artery on postembolization angiography. The clinical success of TAE was defined as bleeding-free survival for one month after TAE. Clinical failure was defined as the need for additional treatment for hemostasis, including repeat TAE or surgery. Using the Cardiovascular and Interventional Radiological Society of Europe (CIRSE) classification system, TAE complications were evaluated [9].

## Results

Embolization procedures

**Figure 1:**
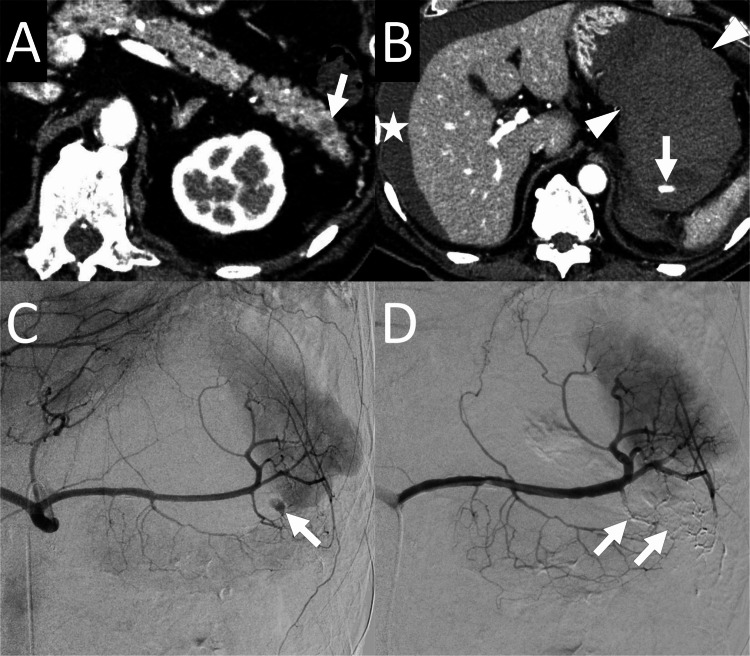
74-year-old man who underwent transcatheter arterial embolization for bleeding caused by endoscopic ultrasound-guided fine-needle aspiration (EUS-FNA) for a pancreatic tail lesion (patient number 4) A: An axial arterial-phase contrast-enhanced computed tomography (CECT) image obtained before EUS-FNA reveals a hypodense lesion (arrow) in the pancreatic tail. B: An axial arterial-phase CECT obtained after EUS-FNA reveals contrast-media extravasation (arrow) near the splenic hilum. A large hematoma (arrowheads) surrounding the extravasation and bloody ascites (star) are observed. C: Angiography of the celiac artery reveals a pseudoaneurysm (arrow) in the caudal pancreatic artery. D: Angiography of the splenic artery after embolization of the caudal pancreatic artery with N-butyl cyanoacrylate (NBCA) reveals no opacification of the pseudoaneurysm. NBCA cast (arrows) is observed in the caudal pancreatic artery.

**Figure 2:**
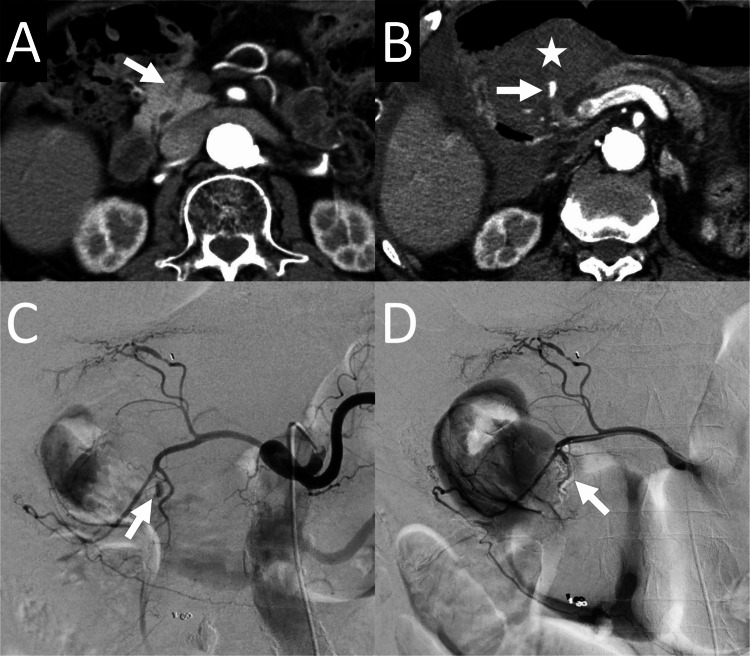
83-year-old man who underwent transcatheter arterial embolization for bleeding caused by endoscopic ultrasound-guided fine-needle aspiration (EUS-FNA) for a pancreatic head lesion (patient number 6) A: An axial arterial-phase contrast-enhanced computed tomography (CECT) image obtained before EUS-FNA reveals a slightly hypodense lesion (arrow) in the pancreatic head. B: An axial arterial-phase CECT obtained after EUS-FNA reveals contrast-media extravasation (arrow) near the pancreatic head. A hematoma (star) surrounding the extravasation is observed. C: Angiography of the celiac artery reveals a pseudoaneurysm (arrow). Although the location of the pseudoaneurysm is not clear from the image, the pseudoaneurysm was in the anterior superior pancreaticoduodenal artery (ASPDA). D: Angiography of the gastroduodenal artery after embolization of the ASPDA with N-butyl cyanoacrylate (NBCA) reveals no pseudoaneurysm opacification. NBCA cast is observed in the pseudoaneurysm and the posterior superior pancreaticoduodenal artery due to backflow (arrow).

In all patients, TAE was performed under local anesthesia and moderate sedation (Figures 1, 2). Via the transfemoral approach, a 4- or 5-F diagnostic catheter (shepherd hook, hook, or Rösch celiac) was used to cannulate the celiac or the superior mesenteric arteries. A microcatheter with a distal tip of 1.7-1.9-F was advanced from the diagnostic catheter to embolize the pseudoaneurysm or stop contrast-media extravasation. The embolic agents used were N-butyl cyanoacrylate (NBCA) (n = 2), NBCA and coils (n = 2), coils (n = 1), and gelatin sponges (n = 1). At a ratio of 1:1.5-3, NBCA was mixed with lipiodol. The details of the embolization procedure are presented in Table 2.

**Table 2:** Embolization procedure and results of embolization ASPDA: anterior superior pancreaticoduodenal artery; LGA: left gastric artery; NBCA: N-butyl cyanoacrylate; PSPDA: posterior superior pancreaticoduodenal artery

Cases	Angiography findings	Embolized arteries	Embolic agents	Technical success	Clinical success	Complications
1	No pseudoaneurysm or extravasation	LGA	Gelatin sponge	Yes	Yes	No
2	Extravasation	PSPDA	Coils	Yes	Yes	No
3	Pseudoaneurysm	PSPDA	Coils, NBCA	Yes	Yes	No
4	Pseudoaneurysm	Caudal pancreatic artery	NBCA	Yes	Yes	No
5	Pseudoaneurysm	LGA	Coils, NBCA	Yes	Yes	No
6	Pseudoaneurysm	ASPDA	NBCA	Yes	Yes	Duodenal ulcer

Technical and clinical success

Technical and clinical success were achieved in all patients. One complication, a duodenal ulcer, developed in one patient. Bleeding from the duodenal ulcer was observed 14 days after TAE. It was successfully managed conservatively and categorized as grade 2 based on the CIRSE classification system.

## Discussion

The frequency of bleeding caused by EUS-FNA is reportedly ≤2%, with minimal bleeding observed at the puncture sites [4]. Although bleeding resolves spontaneously in most cases, massive bleeding may occur in some [5,10]. Two studies based on a nationwide database have reported the frequency of severe bleeding requiring transfusion caused by EUS-FNA for pancreatic masses (7/3090; 0.23%) and that for gastrointestinal submucosal tumors (1/1135; 0.09%) [11,12]. In both studies, bleeding was managed endoscopically, and none of the patients required TAE. Another study reported the frequency of bleeding requiring transfusion caused by EUS-FNA in 22 tertiary centers (5/13566; 0.037%) [13]. However, the frequency of TAE was not mentioned in that study. So far, only a few cases needing TAE for bleeding caused by EUS-FNA have been reported. One study reported a case of massive bleeding immediately after EUS-FNA for a pancreatic head mass [5]. TAE was performed for an inferior pancreaticoduodenal artery pseudoaneurysm; however, the patient died a few days later. Another study reported a case of bleeding complications ten days after EUS-FNA for a pancreatic head mass [6]. TAE was performed for a gastroduodenal artery branch pseudoaneurysm, and the bleeding was successfully controlled. In the present study, TAE was needed for bleeding in six cases of the 4217 EUS-FNA procedures (0.14%). Since EUS-FNA is performed for artery-rich organs, including the pancreas, the risk of arterial injury requiring hemostasis by TAE is inevitable. The reason for the lack of need for TAE to control bleeding in the two studies evaluating the frequency of bleeding based on a nationwide database remains unknown.

In this study, a duodenal ulcer developed in one patient (16.6%). Gastrointestinal ulcers are a complication of TAE for gastrointestinal bleeding [13,14]. We believe that the duodenal ulcer occurred due to ischemia caused by the NBCA injection for the anterior superior pancreaticoduodenal artery pseudoaneurysm. Considering that TAE was performed immediately for life-threatening bleeding, the complication rate was acceptable.

The main limitation of this study was its small sample size, possibly due to the rarity of bleeding caused by EUS-FNA necessitating TAE. Moreover, the small number of case reports on TAE for bleeding caused by EUS-FNA may be due to the underreporting of cases with complications during EUS-FNA. Although the sample size was small, this study demonstrated the effectiveness of TAE for bleeding caused by EUS-FNA. Thus, this study informs physicians and interventional radiologists that, albeit rare, arterial bleeding can occur with EUS-FNA and that TAE is an effective and safe procedure to stop such bleeding.

## Conclusions

Although bleeding caused by EUS-FNA is generally self-limiting, it can occasionally necessitate TAE. In this study, we characterized six patients who underwent TAE for bleeding caused by EUS-FNA. Technical and clinical success were achieved in all patients. However, one patient developed a complication (a duodenal ulcer). This study revealed the effectiveness and safety of TAE for bleeding caused by EUS-FNA.
